# A case report of pyopneumopericardium following bungee jumping in a patient with tuberculosis

**DOI:** 10.1097/MD.0000000000019894

**Published:** 2020-05-15

**Authors:** Yong Zheng Guo, Xiao Feng Li, Qiong Ling Bao, Qun Song, Hai Ying Yu, Ming Jian Zhu, Jun Wei Su, Biao Zhu, Kai Jin Xu, Lan Juan Li

**Affiliations:** a Department of Infectious Diseases, State Key Laboratory for Diagnosis and Treatment of Infectious Diseases, Collaborative Innovation Center for Diagnosis and Treatment of Infectious Diseases, First Affiliated Hospital, College of Medicine, Zhejiang University, Hangzhou; b Huzhou Central Hospital, Huzhou, China.

**Keywords:** bungee jump, purulent pericarditis, *Streptococcus sanguinis*, tuberculosis

## Abstract

**Rationale:**

Pyopneumopericardium related to bungee jumping is a rare occurrence in the current antibiotic era. We present a case of esophagus-seeded *Streptococcus sanguinis* pyopneumopericardium in a young man with tuberculosis who had just completed bungee jumping.

**Patient concern:**

A 27-year-old man was hospitalized with a 1-day history of fever, chest tightness, and intermittent sharp chest pain after bungee jumping for the first time.

**Diagnoses:**

Clinical examinations, thoracentesis, and pericardiocentesis revealed pyopneumopericardium, pyopneumomediastinum, and suppurative pleurisy secondary to bungee-jumping-related traumas. Pericardial fluid cultures were positive for *S sanguinis*, and *Mycobacterium tuberculosis* complex genetic test was positive in both sputum and pleural effusion.

**Interventions:**

The patient improved with drainage and comprehensive antimicrobial therapy.

**Outcomes:**

The patient developed constrictive pericarditis and underwent pericardiectomy after 6 months of anti-tuberculosis treatment. During the 6-month follow-up after surgery, he recovered uneventfully.

**Lessons:**

This case adds to the long list of bungee-jumping complications. Early diagnosis to initiate appropriate therapy is critical for pyopneumopericardium patients to achieve good outcomes.

## 1 Introduction

Pyopneumopericardium is a serious disease of the heart, with rapid progress and a high mortality rate. However, because of the discovery of new antibiotics, it has extremely low morbidity. Pyopericardium usually develops after trauma, iatrogenic treatments, or the spread of suppurative infection from adjacent organs.^[1]^*Streptococcus sanguinis* (*S sanguinis*), a member of the viridans group streptococci, is indigenous to the oral cavity, nasopharynx, and gastrointestinal tract.^[2]^ It is capable of causing infective endocarditis (IE) but rarely associated with the pyopericardium. Here, we present a case of *S sanguinis* purulent pericarditis, complicated with tuberculosis and tuberculous pleurisy, in a 27-year-old man after bungee jumping. Whereas the most commonly reported injuries during bungee jumping are to the eyes, spine, bones, and joints,^[3]^ our patient developed injuries of the right mediastinal pleura, thoracic lymphangion, and esophagus, followed by suppurative pleurisy, pyopneumomediastinum, and finally, pyopneumopericardium. This is an extremely rare case that has not been reported previously in the literature, and the patient has provided informed consent for publication of the case.

## 2 Case report

A 27-year-old Chinese man was hospitalized with a 1-day history of high fever, chest tightness, and intermittent breathing-independent sharp right chest pain. The previous day, the patient had performed his first bungee jump from a height of 50 meters. The apparatus performed as expected. Two years previously, he was diagnosed with pulmonary tuberculosis and tuberculous pleurisy and underwent anti-tuberculosis treatment. The patient had a 10-year history of smoking.

Vital signs on admission showed a temperature of 37.7°C, heart rate of 116 beats/min, blood pressure of 128/97 mmHg, respiratory rate of 20 breaths/min, and normal oxygen saturation. Lung auscultation showed that his right lung field breath sounds were diminished in intensity. Cardiac auscultation demonstrated a regular rhythm with no murmur, rub, or gallop. Remaining physical examination was unremarkable. Laboratory analysis showed an elevated white blood cell count (WBC) of 19.4 × 10^9^ cells/l with 81.6% neutrophils, and a high-sensitivity C-reactive protein (hsCRP) of 248.4 mg/l. Liver function tests showed no abnormalities. Computed tomography (CT) of the thorax revealed lung infection, mild pneumomediastinum, and right encapsulated pleural effusion. His initial symptoms pointed to community-acquired pneumonia. Empiric antibiotic treatment was initiated with azlocillin, an ampicillin-derived antibiotic (5.0 g every 12 hours).

Ultrasound-guided thoracentesis performed on day 2 of hospitalization yielded yellow purulent fluid without obvious odor. The WBC count in the pleural effusion was 2.0 × 10^9^ cells/l with 60% polymorphonuclear neutrophils. On day 5, distant heart sounds were auscultated. An urgent transthoracic echocardiography (TTE)-guided pericardiocentesis indicated pyopericardium and yielded 300 ml of yellow purulent fluid. The WBC count in the pericardial effusion was 216.2 × 10^9^ cells/l with 70% polymorphonuclear neutrophils. Chylous tests were positive in both the pleural and pericardial effusions. On pathogen detection, there was no important finding. On day 7 of the hospitalization, the patient's vital signs, general physical condition, and the levels of WBC, high-sensitivity C-reactive protein, procalcitonin, creatinine, and aminotransferase were worse than those before treatment.

**Figure 1:**
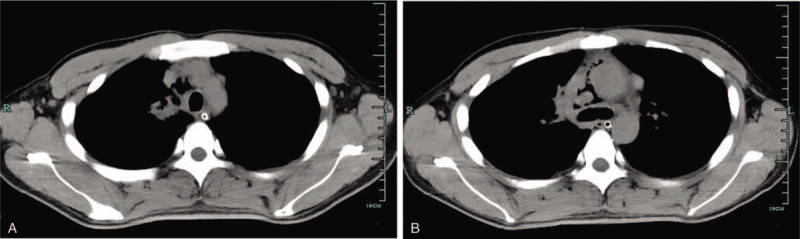
Thoracic computed tomography showing pneumopericardium, pneumomediastinum, and cavernous communicated lesions adjacent to the right superior mediastinum.

On day 8, the patient was transferred to our hospital, a top medical center in the Yangtze River Delta region. Chest auscultation revealed moist rales over the right upper lung, weakened breath sound over the right lower lung, and distant heart sounds. A repeated CT scan of the chest confirmed pyopneumopericardium, pyopneumomediastinum, suppurative pleurisy, and cavernous communicated lesions adjacent to the right superior mediastinum (Fig. 1A-B). Infective endocarditis was ruled out by a repeated TTE. Gram staining of the pericardial effusion showed Gram-positive cocci. A genetic test for *Mycobacterium tuberculosis* complex was positive in both sputum and pleural effusion.

**Figure 2:**
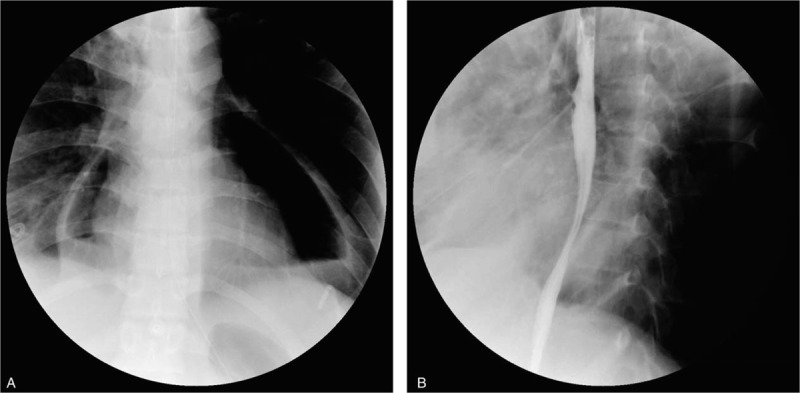
Esophagogram displaying pneumopericardium and a diverticulum in the upper middle part of the thoracic esophagus, with no leak or fistula.

**Figure 3:**
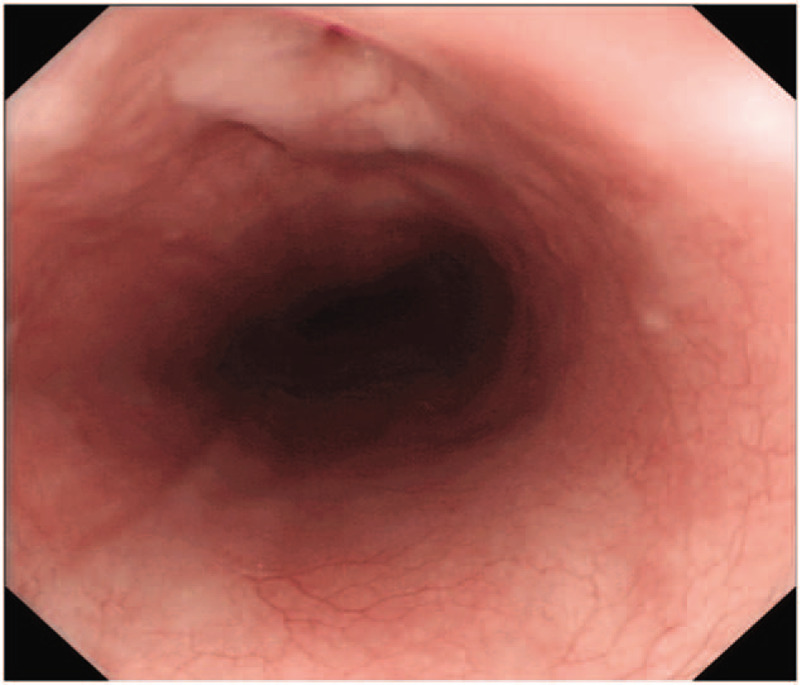
Gastroscopy illustrating two sinus-like changes in the esophagus, 30 cm from the incisors.

Intravenous linezolid (600 mg every 12 hours) was initiated along with meropenem (500 mg every 12 hours), moxifloxacin (400 mg every 24 hours), and oral ethambutol (750 mg every 24 hours). Methylprednisolone was included in the regimen. The dosages were adjusted according to the dynamic changes in liver and renal function. After the antibiotic therapy was adjusted, the patient's symptoms remitted rapidly. Oral meglumine diatrizoate esophagography revealed pneumopericardium and diverticulum in the upper middle part of the thoracic esophagus with no leak or fistula (Fig. 2A-B). Gastroscopy showed 2 sinus-like changes in esophagus, 30 cm from the incisors (Fig. 3). There was no important finding on fiberoptic bronchoscopy.

Until day 17 of hospitalization, two samples of pericardial fluid culture grew *Streptococcus viridans*, subsequently identified as *S sanguinis* using the Vitek 2 GP identification kit (Vitek 2 GP, bioMérieux VITEK-2, Durham, England). Antimicrobial susceptibility test showed that the pathogen was susceptible to linazolamide (31 mm), vancomycin (21 mm), and levofloxacin (18 mm). The pericardial effusion culture became negative 9 days after the antibiotics adjustment. The total drainage of the pleural effusion and pericardial effusion was 3535 ml and 425 ml, respectively.

As the patient's clinical status improved and repeated blood cultures were negative, the antimicrobial regimen was changed to oral moxifloxacin (400 mg once daily), faropenem (200 mg thrice daily), ethambutol (750 mg once daily), and isoniazid (300 mg once daily). The patient was discharged in excellent condition after 6 weeks of hospitalization. One month after discharge, he complained of dyspnea, and subsequent TTE revealed constrictive pericarditis. Pericardiectomy was performed after 6 months of anti-tuberculosis treatment. During the 6-month follow-up after surgery, the patient recovered uneventfully.

## 3 Discussion

Although associated with a variety of bodily injuries, bungee jumping has been growing in popularity among young people since the 1980s. The most frequently reported injuries are ophthalmologic, particularly retinal hemorrhage.^[3]^ Additionally, orthopedic injuries are common, especially bone fracture and joint dislocation. Subdural hematoma, pneumothorax, and pulmonary hemorrhage were reported.^[3]^ To our knowledge, pyopneumopericardium caused by mediastinal infection immediately following esophageal and mediastinal pleural injuries, which were caused by bungee jumping, was not reported previously in the literature.

Our patient had a 10-year history of smoking and a 2-year history of tuberculosis and tuberculous pleurisy. Although, after the initial diagnosis he had completed 6 months of anti-tuberculosis treatment, he did not accept routine follow-up. The thoracic CT scan showed scattered lung infections and right pleural thickening. Additionally, we detected *M tuberculosis* nucleic acid in his sputum and pleural effusion. All these findings suggest that the patient might have had structural lung and pleural lesions including pleural effusion before the bungee jump. These defects did not affect the patient's daily activities, but with the force of the bungee jump, they may have easily ruptured.

A form of Valsalva maneuver (holding the breath and tensing the abdominal muscles) during bungee jumping may lead to a sudden rise in intrathoracic pressure.^[4,5,6,7]^ Transient upward axial acceleration can cause inspiratory effect on the chest wall whereas downward acceleration can cause expiratory effect.^[8]^ During a bungee jump, the acceleration phase converts instantaneously to the deceleration phase. Therefore, underlying disease of tuberculosis might combine with the increased intrathoracic pressure and large shear forces generated during the acceleration/deceleration phase of bungee jumping to cause pleural, esophageal, and thoracic lymphatic trauma. Then, *S sanguinis*, usually found in the esophagus, could become abnormally located in the mediastinum causing pyopneumomediastinum and eventually, fatal pyopneumopericardium.^[9]^

Tuberculous pericarditis remains the most common infective pericarditis in developing countries.^[1]^ Bacterial pericarditis, especially suppurative pericarditis, is very rare in the current antibiotic era. Gram-positive organisms (mostly *Staphylococcus aureus* and *Streptococcus pneumoniae*) dominate the spectrum of bacterial pericarditis etiology.^[10]^ Bacterial pericarditis caused by Gram-positive cocci usually has one of 2 origins: primary (direct implantation during surgery or trauma and hematogenous dissemination) or secondary (contiguous spread from an intrathoracic infection including extension from a myocardial, cardiac valve, or subdiaphragmatic site).^[1,10]^ In this case, the infection in the pyopericardium might have originated from a mediastinal infection, which was caused by the ectopic bacteria after damage to his esophagus.

*S sanguinis* has been increasingly recognized as an important pathogen of IE on native as well as prosthetic valves and implanted cardiac devices, but it rarely causes pericardial infection.^[11,12]^ In our case, although transesophageal echocardiography was recommended to rule out IE, this procedure was postponed because of the patient's instability at that time. Penicillin is an empirical antibiotic choice for treatment of viridans group streptococci infections. However, the initial response was unsatisfactory, which may be related to the resistance of *S sanguinis* to penicillin.^[13,14]^ In a study of patients at risk of developing IE, the rate of *S sanguinis* not susceptible to penicillin was high (75%).^[13]^

Clinicians may have difficulty in recognizing bacterial purulent pericarditis because of its infrequent occurrence and the frequent absence of classic signs. In the absence of clinical manifestations of tuberculosis, our patient had an insidious presentation with subsequent rapid deterioration. Initial clinical and radiological findings pointed to community-acquired pneumonia, but despite antibiotic treatment, 6 days after admission his condition deteriorated and developed into suppurative pericarditis. Physical examination should be the primary element for timely diagnosis, and an urgent TTE and chest CT scan, in our setting, was easy and important to immediately diagnose life-threatening cardiac tamponade and guide percutaneous pericardiocentesis to improve prognosis. Additionally, when our patient's vital signs were stable, gastroscopy and esophagography were performed to assess the severity of his injuries and optimize the therapeutic regimen.

Treatment of purulent pericardial effusion consists of prompt pericardial drainage procedures and culture-guided antibiotic therapy. Repeated pericardial fluid Gram staining and culture was the most important element in the successful treatment of our patient. Without the positive result of Gram-positive cocci staining and culture of *S sanguinis*, he might have been misdiagnosed with tuberculous pericarditis complicated with chylous pericardial effusion due to the injured thoracic lymphangion. Despite timely pericardiocentesis and appropriate antimicrobial therapy, constrictive pericarditis often complicates the scenario. Our patient presented with constrictive pericarditis after 6 weeks of comprehensive treatments, and underwent pericardiectomy after 6 months of anti-tuberculosis treatment. In addition to pericardiectomy, pericardial fenestration or intrapericardial fibrinolysis also can be adopted to treat purulent pericarditis and prevent constrictive pericarditis.^[15,16]^

A limitation of this study is that we did not perform the penicillin-susceptibility test. In future studies, antibiotics sensitivity of *S sanguinis* isolates will be carried out.

## 4 Conclusion

With the increasing popularity of bungee jumping, health care professionals, especially emergency doctors, may encounter various complications. Doctors should always have a high degree of suspicion to ensure a timely diagnosis and appropriate treatment to prevent adverse events. Although cases of such life-threatening injuries remain rare, a detailed cautionary notification would be beneficial in advising participants who have a specific disease or medical risk. Moreover, it is important to specify the proven risks and include possible adverse effects on future health.

In addition, our case highlights the value of daily physical examination, imaging examination, and repeated pathogen detection. Because of the complex therapeutic regimen, complete understanding and trust of the patient are critical for a favorable outcome.

## Acknowledgments

We thank Editage (www.editage.com) for English language editing. In addition, we would like to thank Dr Hui Pan for the assistance in critical reading of the manuscript and fruitful discussions.

## Author contributions

**Conceptualization:** Yong zheng Guo, Xiao Feng Li, Qiong Ling Bao, Qun Song and Hai Ying Yu

**Supervision:** Biao Zhu, Kai Jin Xu and Lanjuan Li.

**Writing – original draft preparation:** Yongzheng Guo and Xiao Feng Li

**Writing – review and editing:** Ming Jian Zhu and Jun Wei Su
